# Narrative psychiatry: Healing through storytelling

**DOI:** 10.1192/j.eurpsy.2021.1193

**Published:** 2021-08-13

**Authors:** J. Gonçalves Cerejeira, C. Rivera Jiménez, I. Santos Carrasco, C. Capella, E. Rodríguez

**Affiliations:** 1 Psiquiatría, HCUV, VALLADOLID, Spain; 2 Psiquiatría, Hospital Clínico Universitario de Valladolid, Valladolid, Spain

**Keywords:** narrative medicine, Narrative Psychiatry

## Abstract

**Introduction:**

We all have the innate ability to tell our story and the way we do it can determine the impact that each problem has on our lives. Storytelling can play a critical role in psychiatric practice and, from this premise, a new way of practicing psychiatry has recently emerged: narrative psychiatry.

**Objectives:**

The objective is to offer a unified vision of narrative psychiatry, providing details on the historical and academic context of this approach.

**Methods:**

A narrative-type literary review focused on narrative psychiatry will be presented.

**Results:**

Narrative psychiatry is an innovative clinical approach in line within narrative medicine and with a specific subtype of postmodern psychotherapy, the narrative therapy of Michael White and David Epston. This novel way of practicing psychiatry arises from critical movements within the discipline but it is an integrative and collaborative perspective: the position of each problem in the patient’s personal narrative is discussed and different therapeutic proposals are addressed, including for instance psychotropic drugs. This integrative posture gives the narrative psychiatrist enough flexibility to equally integrate the scientific achievements of biological psychiatry and the humanizing component of narrative practice. In this literature review, the key tools proposed by the main narrative psychiatrists worldwide for the narrative clinical interview will be exposed.

**Conclusions:**

Narrative psychiatry is a novel approach that narrows the therapeutic relationship and that puts in evidence the history of resistance of the consultant, healing through its own storytelling.

